# Critical role of LncRNA in sepsis-associated acute kidney injury

**DOI:** 10.3389/fphar.2025.1627253

**Published:** 2025-06-27

**Authors:** Lingjian Jin, Jian Liao, Litong Jin, Chunyan Chen, Feng Yuan

**Affiliations:** ^1^ Department of Infectious Diseases, Linhai Second People’s Hospital of Taizhou, Linhai, Zhejiang, China; ^2^ Department of Nephrology, Jiaxing Hospital of Traditional Chinese Medicine, Jiaxing, China; ^3^ Department of Emergency Medicine, Taizhou Central Hospital (Taizhou University Hospital), Taizhou, Zhejiang, China

**Keywords:** sepsis, lncRNAs, SA-AKI, inflammation, signaling pathway

## Abstract

Sepsis is defined as organ dysfunction resulting from a harmful host response to infection. It can lead to multiple organ dysfunction, with the kidneys being one of the most commonly affected organs, resulting in sepsis-associated acute kidney injury (SA-AKI), which is associated with a high mortality rate. Despite significant advances in the treatment of SA-AKI in recent years, the condition continues to exhibit a high mortality rate. It remains a critical issue and clinical burden that necessitates further research to mitigate both acute and chronic consequences. An in-depth exploration of the pathogenesis of SA-AKI is essential for guiding early diagnosis and treatment. Increasing evidence indicates that long non-coding RNAs (LncRNAs) play a crucial role in SA-AKI, typically functioning as competing endogenous RNAs (ceRNAs) that alleviate the inhibition of downstream target genes by microRNAs (miRNAs), thus regulating downstream signaling pathways and participating in vital cellular biological processes and inflammatory responses. A growing number of studies have reported the involvement of LncRNAs in SA-AKI, highlighting the necessity of summarizing the evidence on this topic through a comprehensive review, as LncRNAs can either promote the onset or inhibit the progression of SA-AKI depending on the underlying mechanisms. This paper reviews the pathophysiological mechanisms contributing to the development of SA-AKI, the pivotal role of LncRNAs in this condition, and their potential as biomarkers and therapeutic targets, aiming to provide theoretical guidance for the study and treatment of SA-AKI.

## Introduction

Sepsis is a prevalent cause of death among hospitalized patients and has been associated with high morbidity and mortality rates for many years, with reported mortality rates ranging from 30% to 45% in patients who develop sepsis (Fleischmann et al., 2016; Singer et al., 2016). It is defined as a life-threatening organ dysfunction resulting from a dysregulated host response to infection (Singer et al., 2016). Sepsis is characterized by increased inflammation and immunosuppression, and the imbalance between these factors leads to cellular dysfunction and potentially multi-organ failure, thereby resulting in elevated mortality rates (Singer et al., 2016). Clinically, sepsis often affects organs such as the kidneys, lungs, heart, liver, nerves, and blood. Among these, acute kidney injury (AKI) due to sepsis is one of the most common causes of death in septic patients.

AKI encompasses a heterogeneous group of disorders with diverse etiological factors, including hypovolemia, hypoxia, nephrotoxic drugs, urolithiasis, and sepsis. Notably, sepsis is one of the most significant causes of acute kidney injury, with the mortality rate of sepsis-associated acute kidney injury (SA-AKI) being markedly higher than that of patients with acute kidney injury from other etiologies. While the prognosis of SA-AKI has improved due to advancements in antibiotics, hemodialysis, and renal replacement therapy, further research into the pathogenesis of SA-AKI remains crucial for the development of more effective and innovative treatments.

The study of long non-coding RNAs (LncRNAs) has enhanced our understanding of the pathogenesis of SA-AKI. LncRNAs regulate gene expression through various mechanisms, thereby influencing numerous cellular processes and inflammatory responses. Given that LncRNAs are crucial regulators of cellular homeostasis, their dysregulation significantly contributes to cellular and organ damage (Alsaab et al., 2024). Numerous cell death mechanisms, including apoptosis, necrosis, autophagy, and pyroptosis, are consistently activated during the progression of sepsis (Lelubre and Vincent, 2018). LncRNAs can modulate these cellular processes through different mechanisms, playing a vital role in the onset and progression of SA-AKI. Distinct LncRNA subpopulations exhibit dysregulation during the onset and progression of SA-AKI, and selectively modulating their expression may mitigate renal injury. Therefore, targeting specific LncRNAs at various stages of SA-AKI presents a promising therapeutic strategy. Additionally, combining LncRNAs with other molecular markers could enhance diagnostic and predictive capabilities, facilitating clinical translation. This review does not address the current understanding of the clinical diagnosis and treatment of SA-AKI; instead, it focuses on the role and mechanisms of LncRNAs in SA-AKI, which are frequently studied in cellular and animal models. A comprehensive review of these aspects will contribute to our understanding of the pathogenesis of SA-AKI and aid in translating preclinical findings into clinical applications.

## SA-AKI overview and pathophysiology

SA-AKI is typically defined as AKI occurring in the context of sepsis, without the presence of other significant factors that could explain the AKI. It is characterized by a sudden decline in renal function, which is evidenced by elevated serum creatinine (sCr) levels and oliguria (Bellomo et al., 2017; Bagshaw et al., 2008). In intensive care units, sepsis is the predominant cause of AKI, accounting for nearly half of all AKI cases (Sun J. et al., 2019). Due to the complex and unique pathophysiology of sepsis, SA-AKI presents as a syndrome distinct from other forms of AKI. Although the pathophysiological mechanisms underlying SA-AKI are not fully elucidated, it is evident that the harmful inflammatory cascade associated with sepsis plays a significant role in the onset of AKI. The pathophysiology of SA-AKI is complex and multifactorial, encompassing altered intrarenal hemodynamics, endothelial dysfunction, inflammatory cell infiltration of the renal parenchyma, intraglomerular thrombosis, and tubular obstruction by necrotic cells and debris (Zarjou and Agarwal, 2011). The understanding of the pathogenesis of SA-AKI remains limited, and its treatment is challenging due to the intricate nature of its pathophysiological mechanisms, the complexities of clinical research, and various technical and ethical constraints (Rosen and Heyman, 2001). Much of the current knowledge regarding SA-AKI has been derived from animal models of sepsis, *in vitro* cellular studies, and post-mortem analyses of septic patients. Consequently, these findings should be interpreted with caution, as the response to sepsis in animal models may differ significantly from that in humans (Peerapornratana et al., 2019). The selection of stable and appropriate preclinical models can better replicate the complexities of sepsis in patients and more accurately simulate the clinical features of SA-AKI. Cecal ligation and puncture (CLP) procedures and lipopolysaccharide (LPS) injections are among the most commonly utilized methods to induce experimental septic AKI (Kingsley and Bhat, 2016; Hubbard et al., 2005; Dickson and Lehmann, 2019). CLP procedures induce peritonitis by exposing the animal to feces, while LPS administration mimics the excessive inflammation characteristic of early sepsis by activating the immune system (Dickson and Lehmann, 2019).

The conventional view attributes SA-AKI to an overall reduction in renal blood flow and subsequent tubular epithelial cell death (Bellomo et al., 2017; Poston and Koyner, 2019). This perspective is primarily based on the understanding that major causes of AKI, such as hypovolemia, heart failure, and blood loss, are associated with hypoperfusion and shock (Ronco et al., 2019). However, this traditional view has been challenged in recent years by Langenberg, who demonstrated that SA-AKI can occur in a sheep model of Gram-negative infectious shock despite normal or elevated renal blood flow. This finding suggests that a reduction in overall renal perfusion is not a prerequisite for the development of SA-AKI (Langenberg et al., 2006). This assertion is further supported by Meiden et al., who reported SA-AKI in a similar model without changes in renal blood flow, oxygen delivery, or renal histology (Maiden et al., 2016). Moreover, SA-AKI may manifest in the absence of clinical signs of renal hypoperfusion and hemodynamic instability (Langenberg et al., 2006; Prowle et al., 2012). Notably, autopsies of patients who succumbed to sepsis revealed that tissue damage was not as severe as anticipated and was not correlated with functional changes. Furthermore, SA-AKI occurred without significant renal tubular epithelial cell necrosis, indicating that the extent of cellular damage and death does not account for the severity of sepsis-induced organ dysfunction (Takasu et al., 2013). In conclusion, it is increasingly evident that ischemia-reperfusion injury is not the sole mechanism underlying SA-AKI; rather, multiple mechanisms are likely involved. Across different organs, three mechanisms consistently contribute to septic organ injury: inflammation, microcirculatory dysfunction, and metabolic reprogramming. Currently, these three mechanisms are regarded as fundamental contributors to the development of SA-AKI (Peerapornratana et al., 2019).

**FIGURE 1:**
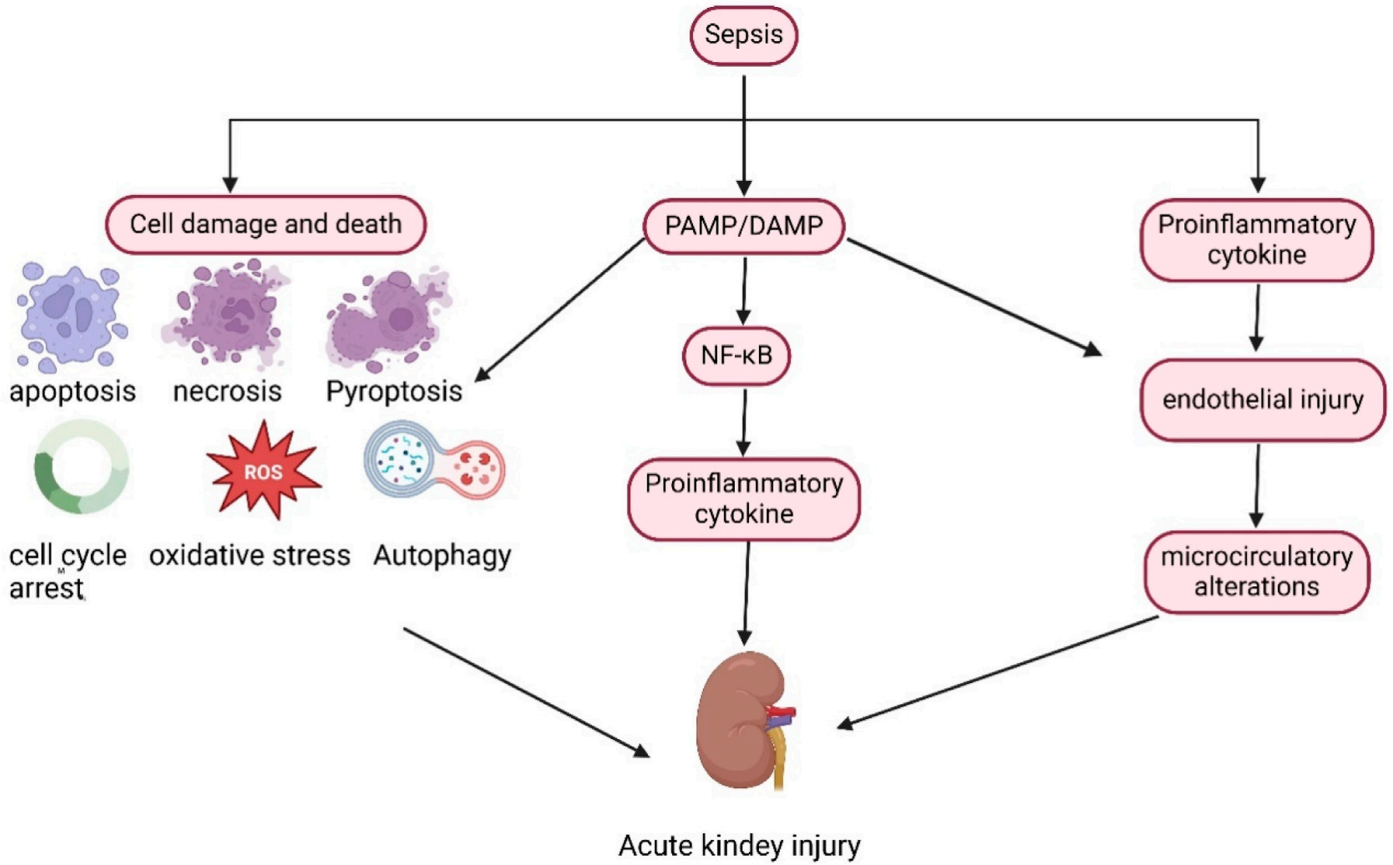
Pathophysiological mechanism of SA-AKI.

In the septic state (Figure 1), inflammatory mediators, including pathogen-associated molecular patterns (PAMPs) and damage-associated molecular patterns (DAMPs), are released into the intravascular space. These molecules bind to membrane-bound pattern-recognition receptors present on the surface of immune cells, such as Toll-like receptors, thereby initiating downstream signaling cascades that lead to the synthesis and release of pro-inflammatory molecules (Peerapornratana et al., 2019). Renal tubular epithelial cells also express Toll-like receptors, particularly TLR2 and TLR4. PAMPs and DAMPs can filter through the glomerulus and recognize TLR2 and TLR4 receptors in the apical membrane of tubular epithelial cells, triggering a downstream signaling cascade that activates nuclear factor kappa beta (NF-κB) to upregulate the expression and release of inflammatory cytokines. This cytokine expression and release lead to an increased inflammatory response, heightened oxidative stress, enhanced production of reactive oxygen species, and mitochondrial damage (Jang and Rabb, 2015; Hotchkiss and Karl, 2003). In the presence of inflammatory factors, endothelial cell damage and glycocalyx shedding trigger the activation of the coagulation cascade, potentially resulting in microthrombus formation and capillary occlusion, which further leads to intrarenal blood shunting and a reduction in glomerular filtration rate (Post et al., 2017; Verma and Molitoris, 2015; Rajendram and Prowle, 2014). Additionally, in response to sepsis and inflammation, cells undergo metabolic reprogramming to optimize and reprioritize energy expenditure, prioritizing cell survival at the expense of cell function. Cell replication is a highly energy-intensive process, and cells may enter cell cycle arrest to prevent energy depletion (Chang et al., 2022; Manrique-Caballero et al., 2021) Furthermore, various cell death processes, including apoptosis, pyroptosis, autophagy, and ferroptosis, directly contribute to sepsis-induced renal injury, with all these factors collectively advancing the progression of SA-AKI.

In the context of sepsis, pathogen-associated molecular patterns (PAMPs) and damage-associated molecular patterns (DAMPs) are released into the circulation, activating the NF-κB pathway and promoting the release of inflammatory factors. Furthermore, sepsis directly induces cellular damage and death. Collectively, these factors contribute to the onset and progression of SA-AKI.

## LncRNAs in SA-AKI

lncRNAs are a significant class of non-coding RNAs, exceeding 200 nucleotides in length, that do not encode proteins during transcription but serve as crucial regulators (Nair et al., 2020; Kopp and Mendell, 2018; Berger et al., 2018). lncRNAs have been demonstrated to regulate gene expression, modulate cellular functions, and influence various physiological and pathological processes at multiple levels. Functionally, lncRNAs are classified as signal transduction, decoy, guide, and scaffold lncRNAs (Wang and Chang, 2011). Mechanistic studies of lncRNAs originated from the discovery of competing endogenous RNAs (ceRNAs), which are not a specific type of RNA but rather a mechanism of RNA interactions that act as sponges for miRNAs, thereby attenuating their inhibitory effects on mRNAs (Guttman and Rinn, 2012; Salmena et al., 2011). lncRNAs are now widely recognized as a regulatory mechanism for ceRNAs. In addition to functioning as ceRNAs, lncRNAs have also been shown to directly regulate gene expression through mechanisms such as DNA methylation, chromatin remodeling, and histone modification. DNA methylation is a crucial epigenetic mechanism that regulates gene expression. This process is catalyzed by DNA methyltransferases (DNMTs), which facilitate the formation of methylation modifications. LncRNAs play a significant role in mediating DNA methylation through various mechanisms, thereby influencing the expression of target genes in both physiological and pathological contexts. Research has shown that lncRNAs can recruit DNMTs, attract TET enzymes (which counteract the DNMT family), isolate or repel DNMTs, modulate DNMT activity, and regulate the expression of DNMTs and TETs, among other mechanisms involved in mediating DNA methylation (Huang et al., 2022). In the context of SA-AKI, it has been demonstrated that lncRNAs regulate the methylation of associated microRNAs, consequently influencing gene expression. For instance, PMS2L2 can upregulate miR-21 by decreasing the methylation of the miR-21 gene (Zhang et al., 2023) Furthermore, lncRNAs can directly interact with proteins (Zhang et al., 2024). They play an essential role in various diseases, including tumors, cardiovascular diseases, inflammatory diseases, and diabetes.

**FIGURE 2:**
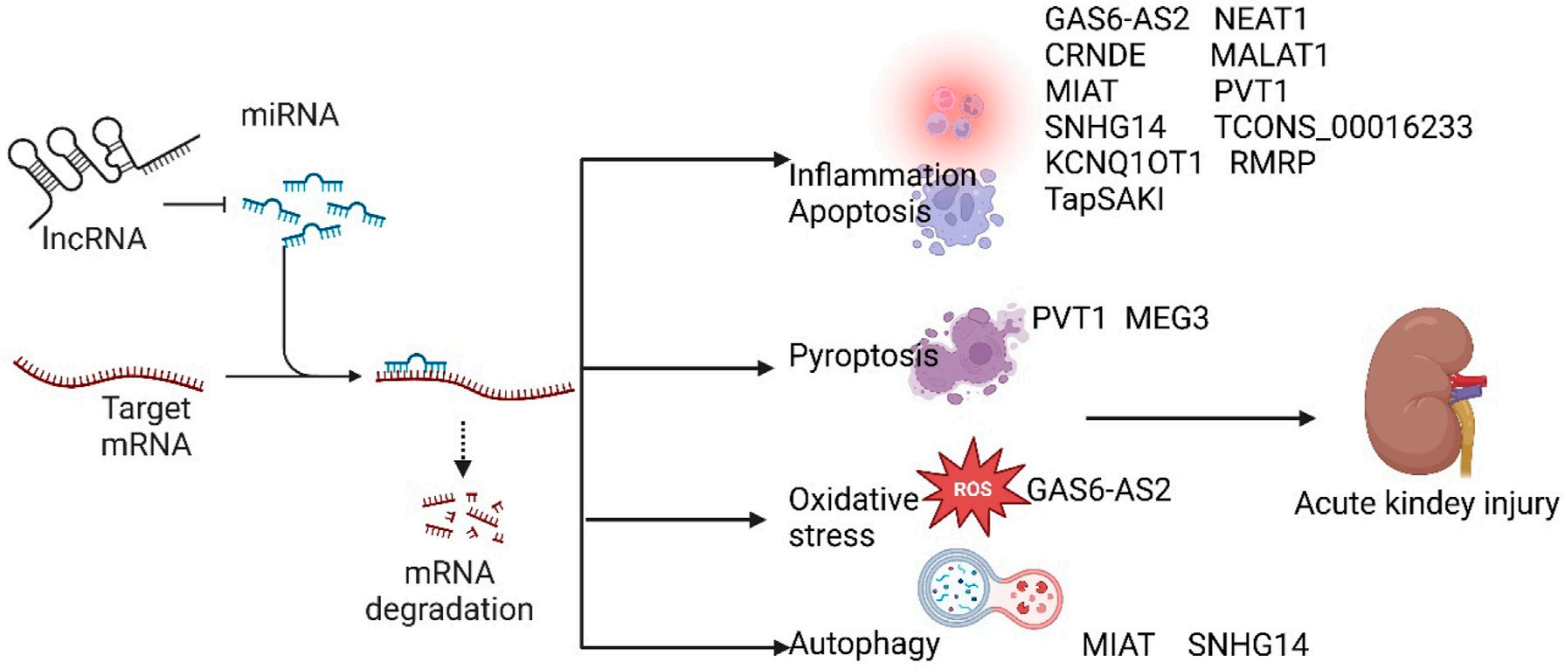
Damaging lncRNAs of SA-AKI promote the development of SA-AKI by inhibiting downstream target microRNAs, regulating downstream related signalling pathways, and inhibiting inflammation, apoptosis, oxidative stress, autophagy, and focal death.

**FIGURE 3:**
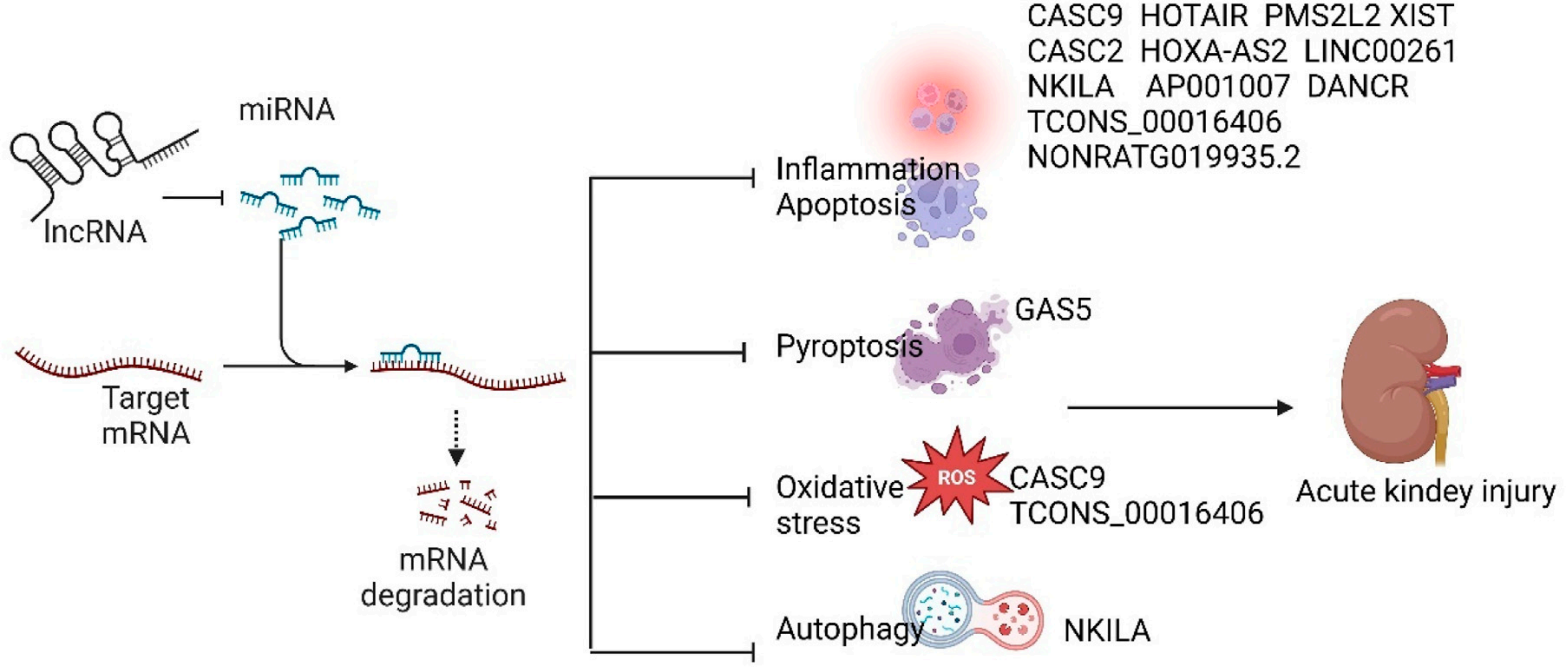
Protective lncRNAs of SA-AKI inhibit the development of SA-AKI by inhibiting downstream target microRNAs and regulating downstream related signalling pathways to inhibit inflammation, apoptosis, oxidative stress, autophagy and focal death.

lncRNAs have been implicated in the pathogenesis of SA-AKI in various studies, and modulation of lncRNAs has been shown to mitigate sepsis-induced acute kidney injury. Based on their roles in SA-AKI, lncRNAs can be categorized into protective and injurious lncRNAs (Table 1 lists the downstream targets and primary functions of various lncRNAs). Protective lncRNAs are typically downregulated in animal and cellular models of SA-AKI, while their overexpression has been associated with improved prognoses by reducing processes such as apoptosis, inflammation, and oxidative stress. Conversely, injurious lncRNAs are often upregulated in these models and tend to exacerbate sepsis-induced acute kidney injury; silencing their expression can alleviate the progression of SA-AKI. In the current study, damaging lncRNAs associated with SA-AKI primarily include NEAT1, CRNDE, MALAT1, PVT1, SNHG14, GAS6-AS2, MIAT, TCONS_00016233, KCNQ1OT1, RMRP, TapSAKI, and MEG3 (Figure 2). Additionally, other damaging lncRNAs encompass CASC9, HOTAIR, PMS2L2, XIST, CASC2, NORAD, HOXA-AS2, LINC00261, NKILA, AP001007, TCONS_00016406, GAS5, NONRATG019935.2, and DANCR (Figure 3). Notably, CRNDE (Sun BQ. et al., 2019; Wang J. et al., 2020)and NORAD (Xie et al., 2022; Lu et al., 2024) have exhibited opposing effects in different studies, which may be attributed to variations in downstream targets and methodological differences; these results warrant careful review. In the current study, it is noteworthy that most mechanistic investigations regarding the role of lncRNAs in SA-AKI have primarily been conducted using animal and cellular models. In contrast, the majority of clinical studies have focused on the expression profiles of lncRNAs in SA-AKI. The lncRNAs that were found to be upregulated in SA-AKI patients include NEAT1, PVT1, SNHG14, MIAT, TCONS_00016233, and RMRP. Conversely, the lncRNAs that were downregulated in SA-AKI patients include PMS2L2, CASC2, HOXA-AS2, LINC00261, AP001007, and DANCR.

**TABLE 1:** Functions of sepsis-associated LncRNAs and their downstream signalling pathways.

LncRNA	Associated miRNAs	miRNA downstream targets	Main findings	Reference
GAS6-AS2	miR-136-5p	XSR1	Promotes apoptosis, inflammation and oxidative stress	Cui et al. (2022)
NEAT1	miR-22-3p	CXCL12	Activates NF-κB signalling pathway and promotes inflammatory response	Feng et al. (2020)
NEAT1	let-7b-5p	TRAF6	Promotes apoptosis, inflammation and inhibits cell proliferation	Gao et al. (2020)
NEAT1	miR-93 - 5p	TXNIP	Promoting LPS-induced HK2 cell injury	Yang et al. (2021a)
NEAT1	miR-125a-5p	TRAF6	Inhibits macrophage M1 polarisation and promotes LPS-induced inflammation	Wang and Guo (2020)
CRNDE	miR-146a	NA	Activation of TLR2/NF-κB pathway to promote inflammatory response and apoptosis	Wu et al. (2020a)
CRNDE	miR-181a-5p	PPARα	Promote cell proliferation, inhibit apoptosis	Wang et al. (2020a)
MALAT1	miR-205	NA	Induction of inflammatory response and apoptosis	Wang et al. (2021a)
MALAT1	miR-370-3p	HMGB1	Induction of inflammatory response and apoptosis, inhibition of cell proliferation	Xu et al. (2020)
MALAT1	NA	NA	Interaction with FUS increases ACSF2 mRNA stability and induces ferroptosis	Duan et al. (2024)
MIAT	NA	NA	Interacts with PTBP1 to activate BECN1-mediated autophagy and promote inflammation and apoptosis	Xu and Zhou (2023)
PVT1	miR-17-5p	NA	Activates NF-κB pathway to promote inflammatory response and apoptosis	Yuan et al. (2021)
PVT1	miR-20a-5p	NLRP3	Promotion of cellular pyroptosis and expression of inflammatory factors	Deng et al. (2021a)
PVT1	miR-27a-3p	OXSR1	Regulation of inflammatory cytokine secretion, cell proliferation and apoptosis	Yang et al. (2022)
SNHG14	miR-495-3p	HIPK1	Regulation of the NF-κB pathway to promote inflammatory cytokines and apoptosis	Yang et al. (2021b)
SNHG14	miR-373-3p	ATG7	Promotion of NF-κB signalling pathway and inflammatory cytokine production, promotion of autophagy	Yang et al. (2024)
TCONS_00016233	miR-22-3p	AIFM1	Promotes apoptosis and inflammatory responses	Zhang et al. (2020)
KCNQ1OT1	miR-212-3p	MAPK1	Activation of p38/NF-κB promotes inflammatory response and apoptosis aggravates acute kidney injury	Wang et al. (2021b)
RMRP	miR-206	DDX5	Activation of NLRP3 promotes LPS-induced apoptosis and inflammatory responses	Zhang et al. (2021b)
TapSAKI	miR-22	NA	Activation of PTEN, TLR4/NF-κB pathways to promote apoptosis and inflammatory responses	Shen et al. (2019)
MEG3	miR-18a - 3P	GSDMD	Promoting cellular pyroptosis and inflammation and exacerbating LPS-induced cellular damage in renal tubular epithelial cells	Deng et al. (2021b)
CASC9	miR-424-5p	TXNIP	Reduces cellular inflammation and apoptosis and enhances cellular antioxidant capacity	Fan et al. (2021)
HOTAIR	miR-34a	Bcl-2	Inhibition of apoptosis and inflammation in renal tissues	Jiang et al. (2019)
PMS2L2	miR-21	NA	Protection of podocytes against LPS-induced apoptosis	Zhang et al. (2023)
XIST	miR-155-5p	WWC1	Suppressed sepsis-induced inflammation and apoptosis	Wang and Cao (2022)
CASC2	miR-155	NA	Inhibition of inflammatory factor secretion, apoptosis and oxidative stress	Wang et al. (2020b)
CASC2	miR-545-3p	PPARA	Inhibited apoptosis, migration, epithelial-mesenchymal transition and oxidative stress	Hu et al. (2021)
NORAD	miR-155-5p	PDK1	Promoting glucose metabolism and protecting renal tubular epithelial cells from LPS damage	Lu et al. (2024)
NORAD	miR-577	GOLPH3	Promotes apoptosis and inflammation	Xie et al. (2022)
HOXA-AS2	miR-106b-5p	NA	Inhibition of Wnt/β-catenin and NF-κB pathways attenuates renal injury in sepsis	Wu et al. (2020b)
LINC00261	miR-654-5p	SOCS3	Inhibits NF-κB activity, thereby inhibiting apoptosis and inflammatory cytokine production	Li et al. (2021)
NKILA	miR-140-5p	CLDN2	Inhibition of apoptosis, autophagy and inflammation	Han et al. (2021)
AP001007	NA	NA	Improves cell viability, protects mitochondrial function, promotes cell survival, and inhibits secretion of pro-inflammatory cytokines	Lin et al. (2024)
TCONS_00016406	miRNA - 687	NA	Inhibits inflammation, oxidative stress and apoptosis by targeting the miR-687/PTEN axis signalling pathway	Liu et al. (2020)
GAS5	miR-579-3p	SIRT1	Activation of the SIRT1/PGC-1α/Nrf2 signalling pathway inhibits cell death and attenuates sepsis-induced kidney injury	Ling et al. (2021)
NONRATG019935.2	NA	NA	Restriction of HuR binding to the 3 ‘UTR region of p53 mRNA reduces HuR-mediated p53 mRNA stability, thereby inhibiting apoptosis	Ding et al. (2021)
DANCR	miR-214	KLF6	Promoting cell viability and inhibiting apoptosis and cytokine production in LPS-treated HK-2 cells	Zhao et al. (2020)

## Mechanism of LncRNA-mediated SA-AKI

### Inflammation

The inflammatory response serves as a fundamental mechanism in the development of SA-AKI, which is intricately linked to the activation of inflammatory signaling pathways and the secretion of pro-inflammatory cytokines. NF-κB is a principal regulator of the inflammatory response and is associated with various diseases. NF-κB is composed of the dimeric subunits p50 and p65, along with the inhibitory subunit IκB (inhibitor of κB). When IκB kinase catalyzes the degradation of the IκB protein, the activated NF-κB forms a dimer that translocates into the nucleus to regulate gene expression, thereby promoting inflammation. The rapid activation of NF-κB, following the degradation of IκB, facilitates its dimerization and subsequent translocation to the nucleus, where it binds to the promoters of several genes, regulating their expression and contributing to the production of inflammatory factors (Sun, 2017; Lawrence, 2009). Moreover, the activation of NF-κB signaling has been associated with various LncRNAs, and reducing NF-κB activity has been suggested to have a positive impact on septic acute kidney injuries. NEAT1 is a well-studied lncRNA in the context of SA-AKI. Several studies have demonstrated that NEAT1 is significantly upregulated in sepsis-induced AKI in both cellular and mouse models. Silencing NEAT1 enhances cell viability and attenuates inflammatory responses (Zhou et al., 2022). Mechanistically, NEAT1 acts as a sponge for miR-22-3p, thereby alleviating the inhibition of its downstream target, CXCL12, and activating the NF-κB pathway. This activation exacerbates the inflammatory response and cellular damage, ultimately promoting the progression of SA-AKI (Feng et al., 2020). Additionally, let-7b-5p has been identified as a target of NEAT1; in HK2 cells, NEAT1 regulates the expression of TRAF6 through the adsorption of let-7b-5p, which subsequently promotes LPS-induced HK2 cell injury and inflammatory responses (Gao et al., 2020). Yang et al. (Yang J. et al., 2021) reported that NEAT1 regulates TXNIP expression by functioning as a sponge for miR-93-5p, which promotes apoptosis, inflammation, and oxidative stress, contributing to the exacerbation of LPS-induced HK2 cell injury. Furthermore, NEAT1 silencing has been shown to ameliorate LPS-induced inflammation by modulating macrophage M2 polarization (Wang and Guo, 2020). Notably, NEAT1 not only activates NF-κB signaling but is also regulated by it. In SA-AKI, the TLR4/NF-κB pathway has been reported to facilitate the overexpression of NEAT1 and its translocation from the nucleus to the cytoplasm. This translocation allows NEAT1 to bind to the scaffold protein receptor for activated protein C kinase 1 (Rack1), activating NLRP3 inflammatory vesicles and promoting the release of inflammatory mediators and responses (Xue et al., 2024). This suggests that there may be a positive feedback amplification mechanism between NEAT1 and NF-κB, whereby the expression of NEAT1 is regulated by NF-κB, which subsequently promotes the activation of NF-κB signaling. The presence of this positive feedback mechanism implies that during the development of SA-AKI, once one component is activated, it amplifies the signals, promotes the activation of the entire signaling pathway, and rapidly induces an inflammatory response. CRNDE has been identified as an oncogene in various cancers and is highly expressed in tumor cells. In the context of SA-AKI, CRNDE was significantly upregulated, promoting the release of inflammatory factors such as TNF-α, IL-6, IL-8, and IL-1β, which exacerbated LPS-induced cellular inflammatory injury. Mechanistically, CRNDE contributes to the increase in cytokines and inflammatory responses by sponging miR-146a, thereby activating the TLR2/NF-κB pathway (Wu S. et al., 2020). Additionally, silencing CRNDE effectively suppressed the activation of TLR3 and p65 in mouse kidney tissues, suggesting that inhibition of CRNDE reduces sepsis-induced AKI by blocking the activation of the TLR3/NF-κB pathway (Sun BQ. et al., 2019). MALAT1 has previously been shown to promote inflammatory injury in the myocardium through the upregulation of TLR4 (Jia et al., 2019). Xu et al. (Xu et al., 2020) also reported that MALAT1 was elevated in both serum from sepsis patients and in an LPS-induced SA-AKI cell model, and its overexpression further aggravated cellular injury. Mechanistically, MALAT1 sponges miR-370-3p, attenuating the inhibition of the target gene HMGB1 by miR-370-3p, which increases the expression of inflammatory mediators such as TNF-α, IL-6, and IL-1β, thereby promoting cellular inflammatory injury. Importantly, paclitaxel prevented LPS-induced AKI by regulating the MALAT1/miR-370-3p/HMGB1 axis, indicating that paclitaxel may serve as a therapeutic agent to mitigate SA-AKI. Furthermore, resveratrol can improve inflammation and protect against SA-AKI by downregulating MALAT1. In resveratrol-treated sepsis model rats, the expression of MALAT1 was downregulated, the secretion of inflammatory factors in serum was reduced, and renal function was improved (Wang B. et al., 2021). LncRNA PVT1 has been implicated in various human diseases, including SA-AKI. Several studies have demonstrated that PVT1 expression is upregulated in the sera of SA-AKI patients, as well as in animal and cellular models of SA-AKI. Notably, the knockdown of PVT1 has been shown to alleviate sepsis-induced acute kidney injury. Furthermore, some studies indicate that PVT1 functions as a sponge for miR-17-5p, thereby activating the NF-κB pathway, which promotes inflammatory responses and apoptosis, leading to increased cellular damage upon LPS stimulation (Yuan et al., 2021). Additionally, Yang et al. (Yang et al., 2022) reported that PVT1 regulates the expression of OXSR1 by targeting miR-27a-3p, modulating the secretion of inflammatory cytokines, cell proliferation, and apoptosis, and promoting LPS-induced injury in HK-2 cells. Curcumin has been reported to exert a protective effect against SA-AKI, potentially through the inhibition of PVT1 and the JNK/NF-κB pathway. Huang et al. (Huang et al., 2020) found that curcumin treatment reduced serum creatinine (Scr) and blood urea nitrogen (BUN) levels, as well as decreased the expression of PVT1 and JNK/NF-κB pathway proteins in the renal tissues of mice with SA-AKI. In contrast, the overexpression of PVT1 diminished the therapeutic effects of curcumin. Furthermore, SNHG14 has been reported to have oncogenic functions in various malignant tumors (Dong H. et al., 2018). The role of SNHG14 in sepsis-induced AKI has been highlighted in several studies. Yang et al. (Yang N. et al., 2021) found that SNHG14 was highly expressed in the plasma of patients with sepsis combined with AKI. *In vitro* studies revealed that SNHG14 inhibited LPS-stimulated proliferation and autophagy of HK-2 cells while promoting apoptosis and the production of inflammatory cytokines. Mechanistically, SNHG14 acts as a ceRNA that negatively regulates the expression of miR-495-3p, thereby alleviating the inhibitory effect of miR-495-3p on its target gene, HIPK1. Moreover, the SNHG14/miR-495-3p/HIPK1 interaction network regulates the NF-κB pathway and promotes the production of inflammatory cytokines such as TNF-α, IL-6, and IL-2β. In addition, several other LncRNAs have been reported to promote inflammation and exacerbate sepsis-induced AKI. For instance, TCONS_00016233 functions as a ceRNA that inhibits the miR-22-3p-mediated downregulation of the apoptosis-inducing factor, mitochondria-associated 1 (AIFM1). This inhibition subsequently enhances the expression of inflammatory cytokines such as IL-1β and TNF-α (Zhang et al., 2020). Additionally, TapSAKI negatively regulates miR-22 expression, thereby upregulating PTEN and proteins associated with the TLR4/NF-κB signaling pathway, which in turn increases the levels of the apoptotic protein cleaved-caspase-3 and inflammatory factors (Shen et al., 2019). Furthermore, KCNQ1OT1 has been shown to be upregulated in LPS-stimulated HK2 cells; its overexpression leads to heightened apoptosis and inflammatory responses, worsening kidney injury. Mechanistically, KCNQ1OT1 has been identified as a miR-212-3p-regulated ceRNA for mitogen-activated protein kinase 1 (MAPK1), which activates the p38/NF-κB pathway (Wang H. et al., 2021).

In the study of SA-AKI, various protective lncRNAs have been identified, which can attenuate the inflammatory response and ameliorate sepsis-induced acute kidney injury by inhibiting inflammatory signaling pathways and the release of inflammatory factors. CASC2 has been linked to a range of human diseases, where it inhibits cell proliferation, invasion, and metastasis, while promoting apoptosis (Huang et al., 2019). Wang et al. (2020a) reported that the serum expression of CASC2 was significantly lower in sepsis patients compared to healthy subjects, and its levels were negatively correlated with the severity of AKI. Further studies demonstrated that CASC2 inhibited the secretion of inflammatory factors, apoptosis, and oxidative stress. Mechanistically, CASC2 directly binds to miR-155 to inhibit its expression. Additionally, CASC2 can attenuate the NF-κB signaling pathway in SA-AKI (Wang et al., 2020b). Lu et al. (2024) reported that NORAD was significantly downregulated in the renal tissues of SA-AKI, while overexpression of NORAD mitigated LPS-induced cellular damage. Bioinformatics analysis and luciferase assays indicated that NORAD downregulated the expression of miR-155-5p, which inhibits glucose metabolism by directly targeting the glucose-metabolizing enzyme pyruvate dehydrogenase kinase 1 (PDK1). Wu et al. (Wu et al., 2020a) investigated the role of HOXA-AS2 in SA-AKI, revealing that HOXA-AS2 expression was reduced in septic patients, animal models of septic kidney injury, and LPS-stimulated HK-2 cells. Overexpression of HOXA-AS2 alleviated sepsis-induced kidney injury. Mechanistically, HOXA-AS2 inhibited the Wnt/β-catenin and NF-κB pathways by targeting miR-106b-5p, thereby alleviating the inflammatory response and exhibiting a protective effect in SA-AKI. DANCR, a novel lncRNA, has been implicated in the pathophysiology of SA-AKI, as evidenced by the reduced serum levels of DANCR in patients with AKI and in LPS-treated HK-2 cells (Zhao et al., 2020). Furthermore, DANCR was found to enhance cell viability while inhibiting apoptosis and cytokine production in LPS-treated HK-2 cells. Mechanistically, DANCR interacts with miR-214, thereby inhibiting the expression of Krüppel-like factor 6 (KLF6), which is known to promote the expression of inflammatory factors in renal diseases (Rane et al., 2019). Li et al. (2021) reported that the expression levels of LINC00261 in the serum of sepsis patients were significantly lower than those in healthy controls, a finding corroborated by similar results in animal models. Further investigations revealed that the overexpression of LINC00261 increased cell viability and decreased inflammatory cytokine production in LPS-treated HK-2 cells. Mechanistically, LINC00261 functions as a sponge for miR-654-5p, which inhibits NF-κB activity by targeting suppressor of cytokine signaling 3 (SOCS3). In a study screening differentially expressed lncRNAs in LPS-exposed HK-2 cells, Lin et al. (2024) identified AP001007 as a potential regulator of SA-AKI, a conclusion supported by the observation of reduced AP001007 expression in the peripheral blood of sepsis patients. Functional analyses demonstrated that increased AP001007 expression mitigated LPS-induced injury in HK-2 cells, enhanced cell viability, protected mitochondrial function, promoted cell survival, and inhibited the secretion of pro-inflammatory cytokines. Similar protective effects were also observed in human kidney-like organs exposed to LPS.

In summary, LncRNA-mediated inflammatory responses significantly contribute to the development and progression of SA-AKI. Pro-inflammatory LncRNAs can activate relevant signaling pathways, such as NF-κB, which promotes the release of inflammatory factors and exacerbates the progression of SA-AKI. Conversely, protective LncRNAs can inhibit the inflammatory signaling pathways, thereby suppressing the progression of SA-AKI.

### Apoptosis

Apoptosis is the process of programmed cell death triggered by the activation of a series of apoptotic proteins regulated by apoptotic genes. Initially, SA-AKI was thought to result from ischaemia and necrosis of renal tubular cells due to sepsis; however, subsequent animal experiments and human data have demonstrated that most patients with SA-AKI do not exhibit reduced renal blood flow perfusion. Pathological examinations of SA-AKI patients revealed a significant increase in apoptosis of renal tubular epithelial cells, strongly suggesting that apoptosis plays a crucial role in the pathogenesis of SA-AKI (Lerolle et al., 2010; Aslan et al., 2018). Certain lncRNAs are implicated in the pathological processes of SA-AKI by mediating apoptosis. In a study examining the role of lncRNA HOTAIR in acute kidney injury in septic rats (Jiang et al., 2019), levels of TNF-α and IL-1β were elevated, and renal tissue cells exhibited signs of apoptosis compared to controls. Conversely, the introduction of lncRNA HOTAIR mimics significantly improved the renal histopathological morphology in septic rats, leading to a marked decrease in TNF-α and IL-1β levels, as well as reduced apoptosis in renal tissues. Mechanistically, lncRNA HOTAIR inhibited the expression of miR-34a, thereby alleviating the repression of miR-34a on its target gene Bcl-2, which in turn inhibited renal tissue apoptosis and mitigated AKI in septic rats. Additionally, lncRNA PMS2L2 has been identified as an inhibitor of LPS-induced inflammation (Yu et al., 2021). Zhang et al. (2023) reported that plasma levels of PMS2L2 were significantly downregulated in SA-AKI patients. Moreover, miR-21, known to inhibit sepsis-induced renal injury, was also significantly downregulated in the SA-AKI group (Zhang et al., 2021a; Pan et al., 2019). In the plasma of SA-AKI patients, the expressions of PMS2L2 and miR-21 were positively correlated. Mechanistically, PMS2L2 can upregulate miR-21 by reducing the methylation of the miR-21 gene, thereby protecting podocytes from LPS-induced apoptosis (Zhang et al., 2023) Ding et al. (Ding et al., 2021) identified differentially expressed lncRNAs through RNA sequencing in a rat model of SA-AKI. Notably, the expression level of lncRNA NONRATG019935.2 (9,952) demonstrated the most significant reduction in both septic AKI rats and LPS-treated NRK-52E cells. Overexpression of 9,935 inhibited c-caspase three expression in LPS-treated NRK-52E cells and delayed the progression of septic AKI. Mechanistically, 9,935 reduced HuR-mediated p53 mRNA stability by limiting the binding of human antigen R (HuR) to the 3′untranslated region (UTR) of p53 mRNA. This indicates that 9,935 plays a role in septic AKI by inhibiting p53-mediated apoptosis of renal tubular epithelial cells, and that this critical function of 9,935 is contingent upon its disruptive effect on HuR-mediated p53 mRNA stability.

### Oxidative stress

Oxidative stress refers to a state of imbalance between oxidative and antioxidant effects within the body, resulting in the accumulation of reactive oxygen species (ROS). The excessive release of ROS into the cytoplasm leads to the oxidative modification of proteins and lipids, which ultimately causes oxidative damage and severely impacts cellular function. Numerous studies have established that oxidative stress occurs during sepsis-associated acute kidney injury (SA-AKI), and heightened oxidative stress exacerbates SA-AKI. LncRNA GAS6-AS2 has been reported to be upregulated in LPS-treated HK2 cells and in a CLP-induced rat model. The knockdown of GAS6-AS2 resulted in decreased levels of ROS and malondialdehyde (MDA), alongside increased superoxide dismutase (SOD) levels, indicating a reduction in oxidative stress. Furthermore, serum creatinine (sCr) and blood urea nitrogen (BUN) levels were also reduced, leading to the amelioration of renal injury. Mechanistically, GAS6-AS2 acts as a sponge for miR-136-5p, thereby mitigating the inhibitory effect of miR-136-5p on the downstream target gene OXSR1 (Mercier-Zuber and O'Shaughnessy, 2011). OXSR1 is a serine/threonine kinase that regulates downstream kinases in response to environmental stress and is responsible for the co-transport of ions in the kidneys. OXSR1 plays a critical role in influencing the apoptotic process and is a key regulator of both oxidative stress and inflammatory pathways (Chen et al., 2004; Cusick et al., 2006; Qin et al., 2019). Additionally, SNHG14 may contribute to LPS-induced oxidative stress in HK-2 cells, with SNHG14 overexpression further enhancing SA-AKI via oxidative stress (Shi et al., 2021).

In contrast, the overexpression of certain lncRNAs has been shown to inhibit oxidative stress and ameliorate renal injury. CASC9 has been reported to play a protective role in sepsis-induced organ damage (Wang et al., 2020c). Fan et al. (2021) demonstrated that CASC9 was significantly downregulated in a LPS-induced AKI model. Conversely, the overexpression of CASC9 mitigated cellular inflammation and apoptosis while enhancing cellular antioxidant capacity. Mechanistically, miR-424-5p has been identified as a downstream target of CASC9, and the interaction between CASC9 and miR-424-5p promotes the expression of thioredoxin-interacting protein (TXNIP), which has previously been implicated in the regulation of renal oxidative stress in AKI (Wen et al., 2018; Ji et al., 2019). Furthermore, the overexpression of lncRNA TCONS_00016406 alleviates SA-AKI by suppressing oxidative stress. Mechanistically, lncRNA TCONS_00016406 acts as a sponge for miRNA-687, which in turn alleviates SA-AKI by activating the miR-687/PTEN signaling pathway (Liu et al., 2020).

In summary, oxidative stress induces oxidative damage in cells and facilitates the progression of SA-AKI. Modulating oxidative stress by regulating the expression of related LncRNAs may ameliorate sepsis-induced renal injury, suggesting that targeting oxidative stress-related LncRNAs represents a promising strategy for the treatment of SA-AKI.

### Cellular pyroptosis

Scorch death represents a distinct form of regulated cell death, characterized by cell swelling and membrane rupture, which leads to the release of intracellular contents and the activation of potent inflammatory responses (Ding et al., 2016). Cellular pyroptosis is primarily mediated by the activation of inflammatory vesicles. In the context of sepsis and acute kidney injury, the most extensively studied inflammatory vesicle is NLRP3, which is activated in response to stimulation by PAMPs and DAMPs. These stimuli subsequently recruit and activate cysteine aspartic proteases, predominantly caspase-1. The activation of caspase-1 triggers the cleavage of Gasdermin D (GSDMD), a key protein responsible for pore formation in the plasma membrane. The N-terminal fragment of GSDMD oligomerizes to create a pore in the cell membrane, leading to cell lysis and the release of cellular contents. The unique structure of the kidney, particularly the high metabolic demands of renal tubular epithelial cells, renders them highly susceptible to pyroptotic injury (Li et al., 2022). Pyroptosis results in tubular necrosis, impaired filtration, and ultimately renal failure (Wu et al., 2025). In recent years, there has been increasing evidence that cellular juxtaposition plays a key role in the pathophysiology of SA-AKI (Z et al., 2024; Sun et al., 2024; Liu et al., 2025). Cellular juxtaposition in SA-AKI is associated with LncRNAs. Deng et al. (Deng et al., 2021a) reported that PVT1 expression was upregulated and cellular juxtaposition was increased in both animal and cellular models of SA-AKI. Notably, PVT1 knockdown significantly inhibited cellular pyroptosis. Mechanistically, PVT1 functions as a sponge for miR-20a-5p, which directly targets NLRP3, suggesting that PVT1 promotes cellular juxtaposition through the miR-20a-5p/NLRP3 signaling pathway, thereby exacerbating sepsis-induced AKI. Additionally, LncRNA MEG3-mediated juxtaposition of renal tubular epithelium plays a crucial role in the pathogenesis of SA-AKI. Silencing of MEG3 downregulated GSDMD expression and reduced focal cell death. Furthermore, MEG3 regulates the expression of GSDMD by acting as a competing endogenous RNA (ceRNA) for miR-18a-3p, which promotes focal death of tubular epithelial cells (TECs) (Deng et al., 2021b). These studies suggest that certain LncRNAs can promote cellular pyroptosis in SA-AKI and facilitate its progression. Therefore, targeted inhibition of the pyroptosis pathway and related LncRNAs may represent a potential strategy for the treatment of SA-AKI.

In contrast, certain LncRNAs have been shown to inhibit cellular pyroptosis, thereby improving the prognosis of SA-AKI. Hua et al. (Ling et al., 2021) reported that LncRNA GAS5 was downregulated in both *in vitro* and *in vivo* models of sepsis-induced kidney injury. Mechanistically, GAS5 negatively regulates miR-579-3p, which diminishes the inhibitory effect of miR-579-3p on its target gene SIRT1. This interaction further activates the SIRT1/PGC-1α/Nrf2 signaling pathway, leading to the inhibition of cellular focal death and the mitigation of sepsis-induced kidney injury.

### Autophagy

Autophagy is a form of programmed cell death characterized by a self-digestive process in which cells utilize lysosomes to degrade their own damaged, denatured, or senescent macromolecules and organelles, influenced by external environmental factors. Autophagy can provide energy and promote cell survival through a circulatory mechanism under metabolic stress; however, excessive autophagy can lead to autophagic cell death (Chen et al., 2024). Autophagy plays a crucial role in the pathogenesis of SA-AKI, with LncRNAs reported to influence the progression of SA-AKI by regulating autophagy. The expression of LncRNA MIAT has been shown to be upregulated in patients with SA-AKI and in LPS-stimulated HK-2 cells. Downregulation of MIAT attenuated autophagy and enhanced cell viability. Mechanistically, MIAT activates BECN1-mediated cellular autophagy by interacting with PTBP1, and the elimination of BECN1 significantly reverses the effect of MIAT silencing on cell viability (Xu and Zhou, 2023). Additionally, SNHG14-mediated autophagy is associated with SA-AKI; specifically, SNHG14 sponges miR-373-3p and reduces the inhibition of its target gene, ATG7, by miR-373-3p, thereby promoting autophagy and the progression of SA-AKI (Yang et al., 2024). Interestingly, the expression of SNHG14 is regulated by m6A modification, with FTO removing the m6A modification of SNHG14, which decreases its stability and hinders its expression (Yang et al., 2024). In summary, LncRNA-mediated autophagy promotes the progression of SA-AKI, and the inhibition of autophagy and associated LncRNAs presents a promising strategy for the treatment of SA-AKI. However, due to the dual nature of autophagy, its exact role in SA-AKI remains uncertain and requires further investigation.

### Cell proliferation

The rate of cell proliferation is closely correlated with the repair of tissue cells; when normal cell proliferation is inhibited, it tends to exacerbate disease progression. In the context of sepsis, renal cell proliferation is suppressed, thereby worsening AKI. Several lncRNAs regulate cell proliferation, thereby influencing the progression of SA-AKI. For instance, SNHG14 has been shown to inhibit LPS-stimulated proliferation of HK-2 cells (Yang et al., 2021). Additionally, MALAT1 is found to be elevated in both serum and LPS-injured cells from septic patients, where it also inhibits cell proliferation (Xu et al., 2020). Furthermore, the silencing of NEAT1 mitigates the LPS-induced inhibition of cell proliferation (Gao et al., 2020). These findings suggest that cell proliferation is inhibited in the context of sepsis, and that modulation of the relevant lncRNAs can alleviate this inhibition, thereby potentially mitigating the progression of SA-AKI.

### Ferroptosis

Ferroptosis is a novel form of cell death characterized by lipid peroxidation, glutathione depletion (GSH), and the inactivation of glutathione peroxidase 4 (GPX4). Recent studies have emphasized the significant correlation between ferroptosis and SA-AKI. Furthermore, lncRNAs have been shown to regulate iron death, thereby influencing the progression of SA-AKI. One study demonstrated that MALAT1 promotes iron death, thereby accelerating the progression of SA-AKI. Conversely, silencing MALAT1 inhibits ferroptosis and mitigates renal injury. Mechanistically, MALAT1 enhances the stability of ACSF2 mRNA by interacting with FUS. Through its interaction with FUS, MALAT1 contributes to ferroptosis and exacerbates SA-AKI by stabilizing ACSF2 mRNA (Duan et al., 2024).

## The potential of lncRNAs as biomarkers and therapeutic targets in SA-AKI

The occurrence of SA-AKI often leads to delays in effective interventions, primarily due to the absence of reliable early biomarkers. Scr and urine volume, while commonly used, have significant limitations in the early diagnosis of SA-AKI, as their abnormalities typically indicate that more severe renal injury has already transpired. Therefore, the identification of non-invasive biomarkers that are both sensitive and specific for the early diagnosis of SA-AKI is crucial. lncRNAs have emerged as promising transcriptomic biomarkers and have been investigated in various biological samples, including histiocytes, plasma, and urine. Their abundance and stability in body fluids position circulating lncRNAs as potential molecular markers of disease. Recent studies have underscored the potential of lncRNAs as biomarkers for SA-AKI. Several lncRNAs are elevated in the urine and plasma of patients with SA-AKI, indicating their potential as valuable diagnostic biomarkers. For instance, a study assessed the expression profiles of circulating lncRNAs in patients with septic AKI, septic non-AKI patients, and healthy controls. The findings revealed that lncRNA TCONS_00016233 was the most significantly upregulated lncRNA, exhibiting a sensitivity of 71.9% and a specificity of 89.6% for AKI detection (Zhang et al., 2020). Furthermore, lncRNA NEAT1 was found to be elevated in the serum of patients with sepsis-induced AKI compared to controls, showing a positive correlation with the severity of AKI (Zhou et al., 2022). Additionally, the downregulation of protective lncRNAs has been linked to the development of SA-AKI. Zhang et al. (2023) investigated the expression of PMS2L2 in SA-AKI patients, sepsis patients without AKI, and healthy controls. Their results indicated that PMS2L2 was significantly downregulated in the plasma of the SA-AKI group compared to both the sepsis and control groups, with no significant differences observed in PMS2L2 plasma expression levels between the sepsis and control groups (Salmena et al., 2011). This suggests that altered PMS2L2 expression may be specifically implicated in SA-AKI rather than in sepsis alone. Collectively, these studies indicate that lncRNAs could facilitate early detection of sepsis-induced AKI, thereby enabling timely intervention.

Gene therapy, an emerging therapeutic approach, has demonstrated significant potential across various diseases. A critical prerequisite for effective gene therapy is the identification of suitable targets. Numerous studies have indicated that modulating lncRNA expression can mitigate sepsis-induced renal injury, suggesting that selective regulation of lncRNAs may serve as a promising therapeutic strategy for the treatment of SA-AKI. Certain lncRNAs have exhibited encouraging therapeutic effects in animal models of septic acute kidney injury. In the case of detrimental lncRNAs, down-regulating their expression presents a viable therapeutic option. For instance, the *in vivo* knockdown of GAS6-AS2 has been shown to reduce sepsis-induced apoptosis, inflammation, and oxidative stress, lower sCr and BUN levels, and effectively alleviate kidney injury (Cui et al., 2022). Several studies have reported that the silencing of NEAT1 ameliorates sepsis-induced apoptosis, inflammation, and inhibition of proliferation, as well as mitigates kidney injury (Wang and Guo, 2020; Dong et al., 2018b). Furthermore, drugs such as resveratrol and paclitaxel have been shown to attenuate inflammation in AKI by inhibiting MALAT1 expression, thereby improving the prognosis of SA-AKI (Xu et al., 2020; Wang et al., 2021b). Protective lncRNAs can alleviate sepsis-induced renal injury through their upregulation; for instance, overexpression of CASC9 reduces cellular inflammation and apoptosis both *in vivo* and *in vitro*, enhances cellular antioxidant capacity, and diminishes sepsis-induced renal injury (Wang et al., 2020c; Fan et al., 2021). Similarly, overexpression of HOXA-AS2 has been shown to alleviate sepsis-induced kidney injury (Wu et al., 2020b). Although these approaches appear theoretically effective, numerous limitations remain in practical treatment. First, one of the most significant challenges in developing lncRNA-based therapies for SA-AKI is the identification of optimal lncRNA candidates or targets, which necessitates a careful balance between the benefits and drawbacks of lncRNA therapies that may raise concerns regarding their clinical value. Second, lncRNAs delivered into the body often lack tissue-specific targeting and may be absorbed by other tissues, leading to adverse effects. A potential strategy to address this issue involves the development of vectors designed to transport lncRNA to specific tissues; recent advancements in vectors, such as viral particles and nanoparticles, have made targeted transport of lncRNA feasible (Cui et al., 2022). Moreover, current research remains confined to cellular and animal models, with a notable lack of clinical studies; further research is essential to ascertain the efficacy and safety of lncRNA regulation in clinical settings.

## Conclusion and prospects

SA-AKI is a serious and prevalent complication of sepsis, associated with extremely high mortality rates and poor prognoses in septic patients. It presents a higher mortality rate and exhibits unique and complex pathophysiological mechanisms compared to other causes of AKI. Although several studies have indicated that inflammatory processes and cell death events are critical factors in the development and progression of SA-AKI, the underlying molecular mechanisms remain poorly understood. Advances in molecular biology have facilitated a deeper exploration of the pathological mechanisms associated with SA-AKI, revealing that dysregulated expression of lncRNAs is linked to the occurrence and progression of this condition, suggesting their potential as both biomarkers and therapeutic targets. Nonetheless, current research is not without limitations. For instance, most studies have been conducted at the cellular level and in animal models, raising concerns about the applicability of findings to humans due to differences in genetic composition and signaling pathways. Thus, the efficacy of therapeutic targets that have shown success in animal experiments in human subjects remains to be established. Furthermore, lncRNAs interact with multiple targets and influence various downstream signaling pathways; prior interactions within these pathways may affect other genes, potentially complicating the achievement of specific therapeutic targets. Additionally, the immune response and safety implications of introducing lncRNAs into the human body warrant careful consideration. Despite these challenges, addressing these issues could pave the way for therapeutic strategies targeting lncRNAs, which may prove beneficial not only for SA-AKI but also for the treatment of sepsis-related diseases. In conclusion, the utilization of lncRNAs as therapeutic targets for SA-AKI represents a promising strategy; however, further studies are required to confirm their safety and efficacy. Our understanding of this field remains limited, necessitating additional research to enhance our knowledge.
